# Determining the Effectiveness of Fibrin Sealants in Reducing Complications in Patients Undergoing Lateral Neck Dissection (DEFeND): A Randomised External Pilot Trial

**DOI:** 10.3390/cancers15205073

**Published:** 2023-10-20

**Authors:** Mandeep S. Bajwa, Richard Jackson, Jagtar Dhanda, Catrin Tudur Smith, Richard J. Shaw, Andrew G. Schache

**Affiliations:** 1 Liverpool Head & Neck Centre, Department of Molecular and Clinical Cancer Medicine, The University of Liverpool, William Henry Duncan Building, 6 West Derby Street, Liverpool L7 8TX, UK; 2 Liverpool Clinical Trials Centre, The University of Liverpool, Liverpool L69 3BX, UK; 3 Head & Neck Unit, Liverpool University Hospitals NHS Foundation Trust, Aintree Hospital, Liverpool L9 7AL, UK; 4 Head & Neck Unit, Queen Victoria Hospital NHS Foundation Trust, Holtye Road, East Grinstead, West Sussex RH19 3DZ, UK; 5 Institute of Population Health, The University of Liverpool, Waterhouse Building, Block B, Brownlow Street, Liverpool L69 3GF, UK

**Keywords:** fibrin tissue adhesive, neck dissection, head and neck neoplasms, feasibility studies, clinical trials as topic

## Abstract

**Simple Summary:**

This study assessed the feasibility of surgical trial comparing neck dissection procedures with and without fibrin sealant and whether the proposed trial design was effective. It demonstrated the benefits of pilot/feasibility work prior to a definitive trial. The study concluded that primary outcomes in Head & Neck surgical trials benefit from being pragmatic. Furthermore, research sites without established trials infrastructure require more time to open. Surgeon credentialling is a vital quality assurance step in most surgical trial designs. Fibrin Sealant improved most clinical outcomes assessed but the signal was weak. Therefore, a decision was made not to progress to the definitive trial.

**Abstract:**

Objectives: High-quality randomised controlled trials (RCT) to support the use of Fibrin Sealants (FS) in neck dissection (ND) are lacking. The DEFeND trial assessed critical pilot/feasibility questions and signals from clinical outcomes to inform a future definitive trial. Patients and Methods: The study design piloted was a blinded surgical RCT. All participants underwent unilateral ND for head and neck cancer. Interventional arm: ND with application of FS. Control arm: ND alone. Feasibility outcomes included recruitment, effectiveness of blinding, protocol adherence and evaluating administrative processes. Clinical outcomes included surgical complications (primary outcome), drainage volume, time to drain removal, length of hospital stay, pain and the Neck Dissection Impairment Index. Results: Recruitment completed ahead of time. Fifty-three patients were recruited, and 48 were randomised at a rate of 5.3 patients/month. Blinding of patients, research nurses and outcome assessors was effective. Two protocol deviations occurred. Two patients were lost to follow-up. The mean (SD) Comprehensive Complication Index in the interventional arm was 6.5 (12.8), and it was 9.9 (14.2) in the control arm. The median (IQR) time to drain removal (days) was shorter in the interventional arm (2.67 (2.42, 3.58) vs. 3.40 (2.50, 4.27)). However, this did not translate to a clinically significant reduction in median (IQR) length of hospital stay in days (intervention: 3.48 (2.64, 4.54), control: 3.74 (3.11, 4.62)). Conclusion: The proposed trial design was effective, and a definitive surgical trial is feasible. Whilst there was a tendency for FS to improve clinical outcomes, the effect size did not reach clinical or statistical significance. (ISRCTN99181100).

## 1. Introduction

Neck Dissection (ND) is one of the most frequently performed procedures in head and neck surgical oncology [1]. Fibrin Sealants (FS) are commercially available products that have been investigated across several areas of surgery [2]. FS is applied to the raw surfaces of the surgical wound before closure. They work by replicating the final stages of the clotting cascade. The haemostatic and adhesive qualities are thought to promote surgical site healing. Evidence from a systematic review and meta-analysis on the use of FS in soft-tissue head and neck surgery found they tend to reduce complications and the volume of wound drainage, thereby potentially minimising the retention time of surgical drains [3].

Pilot/feasibility studies have a vital role in effectively developing surgical randomised controlled trials (RCT). Not only do they inform the optimal design of a definitive trial, but more importantly, they identify problems that would prevent the successful delivery of the trial [4]. The latter point is essential in avoiding research waste. While their findings may not directly answer the clinical question, they are relevant to developing the collective understanding of surgical trial design and delivery, a frequently misunderstood point. The results of a pilot/feasibility study can thus inform the design of surgical trials across the boundaries of specialties and diseases.

Drawing upon available evidence, an RCT to determine the effectiveness of FS in reducing complications in patients undergoing lateral ND was indicated. In recognition of the challenges in delivering surgical trials [5,6], an external pilot trial was conducted to address questions on how well components of the proposed study design would work together and determine the feasibility of a definitive trial. Further details on the rationale can be found in the accompanying and prospectively published protocol paper (https://doi.org/10.1186/s40814-020-00618-w accessed on 13 October 2023) [7].

The objectives were as follows:

(1) Ensure recruitment at a rate of four patients/month;
(2) Determine the effectiveness of the blinding strategy;
(3) Ensure randomisation, allocation concealment and data management worked well within the trial;
(4) Assess adherence to the conditions of the protocol;
(5) Provide clinical evidence to inform the sample size.

The decision to progress to a definitive trial was based on recruitment outcomes, the safety of the intervention, sample size estimation and signal following informal assessment of clinical outcomes.

## 2. Patients and Methods

This manuscript has been written in keeping with the CONSORT statement 2010: extension to randomised pilot and feasibility trials [8]. A completed checklist can be found in Supplementary Data S1. The study was prospectively registered with the ISRCTN registry. ISRCTN99181100 was assigned on 16 May 2018. Ethical approval was granted on 14 June 2018. The study was registered with the UK Clinical Research Network study portfolio (Protocol Number: 37896). A comprehensive account of the trial protocol was published prospectively. It is recommended that readers familiarise themselves with this protocol publication [7].

### 2.1. Trial Design

The design piloted was a blinded RCT at two UK tertiary hospitals (Site 1 and Site 2). Patients were assigned to the interventional arm, which received FS (Artiss, Baxter Healthcare Ltd., Hong Kong) in addition to standard of care (SOC), or the control arm, which received SOC alone. FS was administered using the 2 mL pre-filled double chamber syringe preparation of Artiss. One of the double chambers contains Human Fibrinogen and the synthetic protein Aprotinin; the other chamber contains Human Thrombin and Calcium Chloride Dihydrate. These were mixed at the tip of the double syringe and delivered into the wound as a fine spray driven by medical grade air using the “EasySpray” pressure regulator device per the manufacturer’s instructions (Baxter Healthcare LTD). Artiss was chosen because of its low thrombin concentration that allows surgeons the time to manipulate the tissues before the polymerisation is complete; SOC involved performing the ND and achieving haemostasis without adhesive adjuncts. Patients in both arms had a single size 18F surgical drain placed in the posterior gutter (the dead space posterolateral to the major vessels), exiting the skin inferior to the incision. The wounds were closed with resorbable sutures across the platysma layer and metal clips to close the skin. Additional information on the intervention can be found in the protocol publication [7].

### 2.2. Eligibility Criteria

Inclusion criteria:

- Patients due to undergo lateral ND;
- ND to include a minimum of three levels;
- Patients who have the capacity to consent.
- Exclusion criteria:
- Age < 18 years;
- Bilateral ND;
- Presence of a vascular pedicle for reconstruction;
- Pregnancy or breastfeeding;
- Known hypersensitivity reaction to aprotinin;
- Previous exposure to FS within six months;
- Known allergy to dairy products.

In keeping with a pragmatic trial design, patients with coagulation disorders or those taking anticoagulant or antiplatelet medication were included. Patients who were on immunosuppressive therapy or had previous surgery or radiation to the neck were also included.

Participants were identified through Multidisciplinary Team (MDT) meetings and outpatient clinics at the research sites. They received an explanation of the trial and a Patient Information Sheet (see Supplementary Data S2). Patients who agreed to participate consented before their surgery date. The trial-related activity was scheduled to coincide with their routine clinical visits.

### 2.3. Outcomes

The outcome measures listed in Table 1 were collected during the patient’s hospital stay, at follow-ups 1 and 2 (7–14 days and 28–42 days after surgery), and unscheduled visits. Patients left the trial after follow-up 2, as outlined in the protocol publication [7]. Since this was a pilot trial, no formal effectiveness assessments were performed.

Three further refinements were made to the protocol publication [7].

▪ The Comprehensive Complication Index (CCI) was used in addition to the Clavien-Dindo Classification. CCI is derived from the Clavien-Dindo Classification. It adjusts scores for multiple complications on a scale of 0–100, where 0 is no complication, and 100 is death [9,10]. The Minimal Clinically Important Difference (MCID) for CCI is ten [10];
▪ Qualitative work to determine MCID was unnecessary;
▪ Data on the ‘time for the patient to be declared medically fit for discharge’ was not collected because investigators could not define the timing of these decisions.

### 2.4. Sample Size

It was determined that approximately 50 patients (25 in each arm), recruited at a rate of 30% of all eligible patients, would provide sufficient precision on clinical outcomes to inform the design of a definitive trial.

### 2.5. Randomisation

Randomisation lists were computer generated by a statistician before recruiting the first patient. Patients were randomised using a 1:1 ratio using randomly permuted blocks and stratified by recruiting site.

Upon randomisation, the surgeon received a password-protected link via email, which they opened immediately before wound closure to reveal the allocation. This exact time and date was recorded automatically in the electronic Case Report Form (eCRF) and cross-referenced the surgery’s start and finish times to minimise performance bias.

### 2.6. Blinding

Patients, research nurses (RN) and clinical outcome assessors were blinded to the allocation. Only surgical team members in the theatre knew the allocation but could not assess trial outcomes. The effectiveness of blinding was evaluated using the Bang Blinding Index (BBI) at follow-up 2 [11].

### 2.7. Statistical Methods

The primary analysis was carried out on the complete data set based on the ‘intention to treat’ principle, using descriptive statistics. Continuous data were summarised as medians with interquartile ranges (IQR), while categorical data were summarised as frequencies and percentages. In terms of clinical outcomes, aside from descriptive statistics, informal comparisons between allocated groups were made using the T-Test for differences in means. Data measured at baseline and follow-up were analysed using Analysis of Covariance (ANCOVA) methods, analysing the follow-up data, and including baseline data as an adjusting covariate. If the data were significantly skewed, a square root transformation was utilised to make the variation in data more uniform and easier to analyse.

The CCI and total wound drainage volume (mL) were used as performance metrics to identify a learning effect. From pre-trial interactions with recruiting surgeons, some used FS routinely, whilst others used it infrequently or not at all. Surgeons were classified as established users or learners if they had used FS in ND on less than ten occasions. Five surgeons were classified as established users and eight as learners. A post hoc analysis was performed to identify the impact of any learning effect on a future trial design.

## 3. Results

Table 2 presents the baseline variables across both treatment arms. The distribution was broadly similar. However, notable differences included: the control arm had more patients with a poorer WHO Performance Status; all three patients who had modified radical NDs were in the control arm; the interventional arm had a higher median volume of blood loss.

### 3.1. The Proportion of Eligible Patients Randomised to the Study

**Figure 1:**
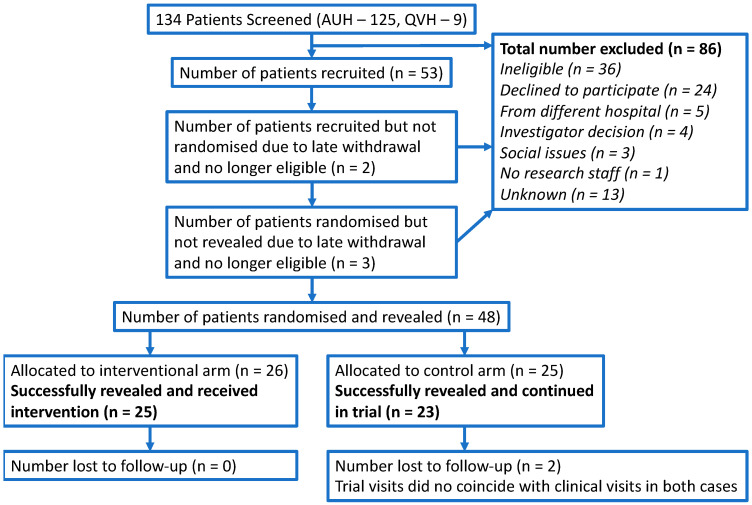
CONSORT Flow Diagram.

The CONSORT flow diagram in Figure 1 summarises the progress through the different phases of the study. Overall, 134 patients were screened, 98 were eligible, 53 were recruited and 48 were successfully randomised and revealed. Therefore, the overall proportion of eligible patients randomised to the study was 49% (48/98). It was predicted that 180 patients would be screened and 30% would be randomised. Whilst the observed number of screened patients was lower than predicted, the proportion of these patients randomised was higher.

### 3.2. Recruitment Rate

**Figure 2:**
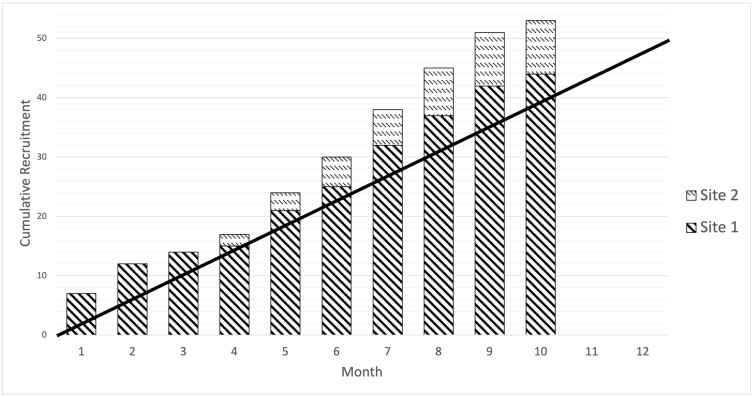
Cumulative recruitment by site. The line represents the predicted recruitment of approximately four patients per month.

The recruitment rate for the study was 5.3 patients/month, which was higher than the target of four patients/month. The recruitment window differed between sites, with Site 1 recruiting over ten months and Site 2 over five months, opening four months after Site 1. The cumulative recruitment curve based on monthly recruitment by the site is presented in Figure 2.

### 3.3. Reasons for Failure to Screen Potentially Eligible Patients

The sites had differing infrastructure to support recruitment for head and neck clinical trials. Site 1 is a large academic centre and screened more patients. Site 2 screened relatively fewer patients; however, a high proportion (66.7%) were randomised and revealed once patients were screened. This was because the clinical pathways for treating patients in Site 2 presented unforeseen barriers. MDT meetings and outpatient clinics in Site 2 were delivered in peripheral (spoke) hospitals, and surgical care was provided centrally (hub). Spoke hospitals were not opened as additional research sites; therefore, patients could not be approached or consented in them. This increased the logistical complexity of screening and recruiting patients before their planned date for surgery.

### 3.4. Reasons for Failure to Randomise Patients

Approximately 22% of eligible patients declined to participate, with a similar rate at both sites (Site 1: 22/98 (22.4%); Site 2: 2/9 (22.2%)). This implies that participants generally accepted the conditions of the trial.

The number of patients randomised was significantly different between sites due to Site 2 taking longer than expected to open the trial, leading to a shorter recruitment window. To avoid further delay, spoke hospitals were not opened. As described in the previous section, this decision came at the price of increasing the complexity of screening and recruiting patients for the trial.

### 3.5. Number of Patients Lost to Follow-Up and the Reason Why

A total of two (4%) patients who were successfully randomised and revealed did not complete follow-up. Both patients were in the control arm, and in both cases, this was because follow-up visits did not coincide with their routine clinical follow-up.

### 3.6. Reasons for Failure to Reveal Allocation at a Specific Time Point

Allocations were revealed during surgery for all patients, with only one exception where the allocation was revealed before the start of surgery due to the recruiting surgeon’s misunderstanding of the protocol. Table 2 shows that the allocation was revealed a median (IQR) of 2.133 (1.55, 2.5) hours after the start of surgery, suggesting good compliance with this aspect of the protocol.

On a separate occasion, trials unit staff were uncontactable to resolve an issue with revealing the allocation during surgery. The recruiting surgeon had forgotten their password and could not reveal the allocation. Because the matter could not be resolved promptly, the patient was empirically placed in the control arm.

### 3.7. Protocol Adherence

In addition to the above protocol deviations related to revealing the allocation, a surgeon documented the allocation in the operative notes on a single occasion, unblinding outcome assessors. All three protocol deviations were managed with targeted education as corrective and preventive actions to resolve compliance issues. These measures were effective in preventing recurrences.

### 3.8. Missing Data

The distribution of missing data between treatment arms is shown in Supplementary Data S3. Overall, there was an even distribution of missing data between treatment arms. Site 2 was noted to have more missing data. Feedback from investigators from Site 2 stated that they found the trial labour intensive. Fewer investigators at the site made the completion of eCRFs in real-time (e.g., for drain volume) challenging.

### 3.9. Fidelity of the Blinding Process

Details of the blinding process assessment are provided in Table 3. There was a tendency for both patients and RNs to believe the patient received the intervention (whether they indeed did or not). In both cases, the BBI and associated 95% CI for the interventional arm indicated a tendency towards ‘unblinding’, and for the control arm, indicated a trend towards ‘negative guessing’. Overall, there was a tendency towards the ‘wishful thinking’ phenomenon, which suggests blinding was effective [11]. Most clinical outcome assessors reported not knowing what treatment patients received and did not attempt a random guess. The BBI and associated 95% CI for the interventional arm indicated a tendency towards ‘unblinding’, and the control arm indicated successful blinding. Overall, this data demonstrated that blinding was effective.

### 3.10. Other Pilot and Feasibility Outcomes

During the study, zero serious adverse events (SAE) were reported, and no patients needed to be unblinded.

### 3.11. Clinical Outcomes

In total, 16 (33.3%) patients experienced at least one complication. Table 4 provides details on the types and severity of complications encountered by the treatment arm. There were more complications in the control arm (14 in control and 10 in interventional arm). However, significant complications that required surgical interventions (grade IIIA and above) were similar across both arms (four in control and five in interventional arm). Of these, there were more haematomas that required a return to theatre in the control arm (three in control and one in interventional arm).

The key clinical outcomes used to informally evaluate the intervention are shown in Table 5. The mean CCI and the median total wound drainage volume were better in the interventional arm. The median time in days to reach the drainage threshold of <1.25 mL/h was the same across both arms. The median time to remove the drain was less in the interventional arm, but this did not translate to a reduction in the median length of hospital stay.

The Neck Dissection Impairment Index (NDII) at baseline was the same across both arms, and patients in the interventional arm scored slightly better at follow-up 2. Pain scores were very low and the same across both arms at every time point. The Wound Healing Questionnaire (WHQ) score was marginally better in the interventional arm.

### 3.12. Post Hoc Analysis of Learning Effect

The results of the post hoc analysis to determine the presence of a learning effect are shown in Table 6. With established users, the intervention appeared to improve CCI and total wound drainage volume, whereas the intervention had the opposite effect in the hands of learners. Furthermore, the SD and IQR suggest that established users had more consistent outcomes. This analysis indicates that a learning effect must be addressed in a future trial design.

### 3.13. Incremental Cost-Effectiveness Ratio

The health economic (HE) analysis and calculations can be found in Supplementary Data S4. The analysis demonstrated that the data was incomplete, and no firm conclusions regarding cost-effectiveness could be made.

## 4. Discussion

These findings represent a detailed narrative of the performance of a head and neck surgical trial designed to assess the effectiveness of FS in ND. Recruitment was completed ahead of time and target, and trial conduct was excellent. Revealing the allocation at a specific time point during surgery was feasible, and quality assurance processes to ensure compliance worked well. The blinding of patients, RNs and clinical outcome assessors was effective. The study produced important nuances and limitations that would improve future trial design.

To summarise the challenges faced by Site 2 in delivering surgical trials outside of large academic centres, the following learning points were identified:

1. Sites without established clinical trial infrastructure need more time to open, and this should be considered in site selection and recruitment planning, especially for trials with narrow recruitment windows;
2. Research sites that recruit patients from MDTs and clinics in spoke hospitals should also open these hospitals to streamline recruitment pathways, though this requires additional set-up time. Patient Identification Centres (PIC) can be a valuable middle ground in the UK as they can identify potentially eligible patients and refer them to research sites for further consideration. They are not allowed to undertake any research activity (e.g., consent patients) and, therefore, are relatively quick to open;
3. Pragmatic outcome measures should be used in head and neck surgical trials, with investigators limiting outcomes to those essential to answering the research question.

Early in the trial, a few protocol deviations were addressed with focused education. None were repeated, but they identified areas to reinforce during site initiation visits. HE data collection was feasible in the hospital setting, but community healthcare resource use and absenteeism from work were not collected, which is a limitation of this study.

Using the CCI rather than the original Clavien-Dindo classification was appropriate as it accounted for the additional morbidity incurred by multiple complications in the same patient. The Clavien-Dindo classification is a generic instrument initially validated in general surgery [12]. Its application can, therefore, be open to interpretation for specific head and neck surgical complications [13]. To overcome the issue of inter-observer variability, the previously published protocol outlined how the classification should be applied to typical head and neck complications [7].

A learning effect associated with using FS in ND was observed, highlighting the importance of having robust quality assurance to preserve the fidelity of the intervention and reduce type II errors. Theatre staff and recruiting surgeons were instructed on the surgical protocol as part of the site initiation process. They were also provided access to video instructions and laminated flow charts were displayed in operating theatres. It is now understood that formal credentialing of surgeons before recruiting patients was necessary.

### Interpretation and Progression to a Definitive Trial

This study emphasises the importance of pilot/feasibility work before a definitive surgical trial, despite the proposed design performing well. It highlighted necessary refinements, challenges, and solutions, enabling a better understanding of the trial. The learning points discussed above can be applied to most surgical trials.

The prespecified criteria for progression included recruitment, safety, sample size estimation and clinical outcome signals. This study demonstrated that a definitive trial could be expected to have excellent recruitment and to be safe for patients. Sample size estimation using CCI as the primary outcome measure, with an MCID of 10 and SD of 11.4 (based on established user results), required 56 patients (28 in each arm) to have a 90% chance of detecting a difference at the 5% level. This is a very similar number of patients recruited for the present study. Importantly, this study was not designed to detect a difference, and the lack of robust quality assurance has introduced a type II error, making any conclusions about effectiveness inappropriate.

## 5. Conclusions

The intervention did demonstrate a positive signal across most clinical outcomes, but effect sizes remained small and did not meet the threshold for clinical significance. Even experienced users did not achieve the MCID of 10 for CCI, and the difference in drainage volume was only 7 mL. With the current sample size and effect sizes, a future trial is unlikely to favour the intervention significantly. This study gives confidence in the proposed trial design for funders but questions the impact of a negative trial on patient care. This is arguably a clinically meaningful finding in its own right. Based on this reasoning, a definitive trial was not pursued. The results and lessons from this study are particularly beneficial to surgical researchers when embarking on future surgical trials across the boundaries of specialties and diseases.

## Figures and Tables

**Table 1:** List of trial outcomes. mL—millilitres, mL/h—millilitres per hour, eCRF—electronic case report form.

DEFeND Trial Outcomes
**Pilot/Feasibility Outcomes**
Reasons for failure to screen potentially eligible patients. Recruitment rate measured as the number of patients randomised each month. Reasons for failure to randomise.Reasons for failure to reveal allocation at a specific time point during surgery. Fidelity of the blinding process as determined by blinding indices. Accuracy of data recording summarised by the number of data items with missing/incomplete data entries. Number of patients lost to follow-up. Protocol adherence measured by the number of major/minor protocol deviations observed throughout the study.
**Clinical outcomes to inform a definitive trial**
Comprehensive Complication Index (CCI). Twice daily wound drainage volume (mL). Time for daily wound drainage volume to reach < 1.25 mL/h as determined by a bespoke algorithm coded into the eCRF. Time to drain removal (dictated by the above algorithm). Total wound drainage volume (mL). Length of hospital stay. Incremental cost-effectiveness ratio.
**Patients reported outcomes to inform a definitive trial**
Neck Dissection Impairment Index (NDII). Daily patient-reported pain score using visual analogue scale (VAS). Wound Healing Questionnaire (WHQ).

**Table 2:** Distribution of baseline and surgical characteristics by treatment arm. RT—radiotherapy, ND—neck dissection, IQR—interquartile range, PT—prothrombin time, aPTT—activated partial thromboplastin time.

Covariate	Level	Interventional Arm	Control Arm	Total
Summary of Baseline Characteristics		N = 26	N = 25	N = 51
Previous Neck Treatment	No Previous Treatment	21 (81%)	21 (84%)	42 (82%)
	Ipsilateral RT	0 (0%)	1 (4%)	1 (2%)
	Contralateral RT	0 (0%)	2 (8%)	2 (4%)
	Ipsilateral ND	2 (8%)	0 (0%)	2 (4%)
	Ipsilateral Open Biopsy	1 (4%)	0 (0%)	1 (2%)
	Other	2 (8%)	1 (4%)	3 (6%)
Height (m)	median (IQR)	1.78 (1.755, 1.828)	1.7 (1.65, 1.78)	1.77 (1.685, 1.795)
Weight (kg)	median (IQR)	84.7 (74.275, 99.55)	71.4 (65.8, 83.4)	81.3 (70.9, 89.5)
Body Mass Index	median (IQR)	27.55 (25.402, 31)	25.94 (24.5, 28.8)	26.91 (24.565, 29.725)
WHO Performance Status	0	19 (73%)	17 (68%)	36 (71%)
	1	7 (27%)	2 (8%)	9 (18%)
	2	0 (0%)	5 (20%)	5 (10%)
	4	0 (0%)	1 (4%)	1 (2%)
Smoking Status	Current	4 (15%)	4 (16%)	8 (16%)
	Ex-Smoker	13 (50%)	8 (32%)	21 (41%)
	Never Smoked	9 (35%)	13 (52%)	22 (43%)
Immunosuppressive Treatment	No	25 (96%)	25 (100%)	50 (98%)
	Yes	1 (4%)	0 (0%)	1 (2%)
Antiplatelet Therapy	No	24 (92%)	24 (96%)	48 (94%)
	Yes	2 (8%)	1 (4%)	3 (6%)
Anticoagulated	No	22 (85%)	23 (92%)	45 (88%)
	Yes	4 (15%)	2 (8%)	6 (12%)
Haemoglobin (g/L)	median (IQR)	143 (133.5, 149)	140 (127.5, 149)	141.5 (128.25, 149)
Platelet Count (10^9^/L)	median (IQR)	228 (175.5, 289)	294 (288.5, 342)	288.5 (210.5, 321)
White Cell Count (10^9^/L)	median (IQR)	6.8 (6.05, 8.75)	8.85 (5.925, 10.3)	7 (6, 9.6)
PT (sec)	median (IQR)	10 (10, 11)	10 (10, 11)	10 (10, 11)
aPTT (sec)	median (IQR)	25 (24, 26)	26 (25, 28)	25 (24, 27)
Randomisation	Randomised but not revealed	1 (4%)	2 (8%)	3 (6%)
	Randomised and revealed	25 (96%)	23 (92%)	48 (94%)
**Summary of Surgical Characteristics**		**N = 25**	**N = 24**	**N = 49**
Primary Resection	No	10 (40%)	11 (46%)	21 (43%)
	Yes	15 (60%)	13 (54%)	28 (57%)
Number of Neck Levels Dissected	0	0 (0%)	1 (4%)	1 (2%)
	3	19 (76%)	16 (67%)	35 (71%)
	4	6 (24%)	4 (17%)	10 (20%)
	5	0 (0%)	3 (12%)	3 (6%)
Cutting Instrument	Cold Steel	15 (60%)	18 (75%)	33 (67%)
	Cutting Diathermy	3 (12%)	2 (8%)	5 (10%)
	Harmonic Scalpel	7 (28%)	3 (12%)	10 (20%)
Intra-operative Blood Loss (mL)	median (IQR)	100 (40, 100)	50 (25, 100)	50 (27.5, 100)
Length of surgery (hours)	median (IQR)	2.467 (1.942, 2.958)	2.05 (1.683, 2.383)	2.2 (1.775, 2.617)
Time to revealing Allocation (hours)	median (IQR)	2.283 (1.667, 2.6)	2.05 (1.358, 2.375)	2.133 (1.55, 2.5)
Time in Recovery Room (hours)	median (IQR)	1.683 (1.3, 2.35)	1.642 (1.417, 1.987)	1.667 (1.367, 2.083)
Nodal Yield	median (IQR)	22 (17.8, 27.5)	22 (19, 28)	22 (18, 28)

**Table 3:** Fidelity of the blinding process. FS—Fibrin Sealant; V—variance estimate, CI—confidence interval.

	Patients		Research Nurses		Clinical Outcome Assessors	
	Interventional Arm	Control Arm	Interventional Arm	Control Arm	Interventional Arm	Control Arm
Strongly believed patient received intervention	10	6	7	5	2	0
Somewhat believed patient received intervention	5	4	12	11	2	2
Somewhat believed patient did not receive intervention	2	0	4	5	1	1
Strongly believed patient did not receive intervention	2	2	0	0	0	1
Did not know/refused to guess	5	9	1	0	19	15
**Bang Blinding Index**	**0.43** **(V = 0.02, 95% CI 0.29–0.57)**	**−0.36** **(V = 0.02, 95% CI 0.22–0.50)**	**0.47** **(V = 0.01, 95% CI = 0.37–0.57)**	**−0.38** **(V = 0.02, 95% CI = −0.52–−0.24)**	**0.22** **(V = 0.01, 95% CI = 0.12–0.32)**	**−0.03** **(V = 0.02, 95% CI = −0.17–0.11)**

**Table 4:** Description of complications and Clavien-Dindo grade by treatment arm. SSI—surgical site infection.

Complication	Interventional Arm		CONTROL ARM	
	Number	Grade	Number	Grade
Neck SSI	1	IIIA	1	II
Other SSI	0		2	II, II
Neck Haematoma	1	IIIB	3	IIIB, IIIB, IIIB
Wound Breakdown	1	I	1	I
Seroma	4	I, I, IIIA, IIIA	3	I, I, IIIA
Chest Infection	1	II	1	II
Other Complications	2	I, IIIB	3	I, I, II
**Total**	**10**		**14**	

**Table 5:** Informal comparison of clinical and patient-reported outcomes by treatment arm. mL—millilitres, SD—standard deviation, IQR—interquartile range, VAS—visual analogue scale.

Outcome	Level	Interventional Arm	Control Arm	*p*-Value
Comprehensive Complication Index	Mean (SD)	6.5 (12.8)	9.9 (14.2)	0.388
Total Drainage Volume (mL)	Natural Scale Median (IQR)	76 (35, 164)	82 (54, 161)	
	Square Root Scale Median (IQR)	8.718 (5.916, 12.806)	9.055 (7.347, 12.680)	0.482
Time to 1.25 mL/h Threshold (days)	Median (IQR)	2.637 (2.625, 3.625)	2.625 (2.620, 3.628)	0.642
Time to Drain Removal (days)	Median (IQR)	2.667 (2.417, 3.576)	3.399 (2.500, 4.266)	0.503
Length of Stay (days)	Median (IQR)	3.476 (2.635, 4.541)	3.735 (3.106, 4.616)	0.479
Neck Dissection Impairment Index	Baseline Median (IQR)	11 (10, 13)	11 (10, 12)	
	Follow-up day 28–42 Median (IQR)	16.5 (13.75, 22.25)	20 (14, 22)	
	Difference Median (IQR)	4.5 (0, 11.5)	7 (2, 11)	0.55
Neck Pain VAS (values out of 10)	Baseline Median (IQR)	0 (0, 1)	0 (0, 1)	
	Follow-up day 7–14 Median (IQR)	1 (0, 2)	1 (0, 3)	
	Follow-up day 28–42 Median (IQR)	1 (0.75, 2)	1 (0, 2)	0.829
Wound Healing Questionnaire	Follow-up day 28–42 Median (IQR)	2 (1, 5)	4 (0, 5)	0.482

**Table 6:** Post hoc analysis to determine the presence of learning effect. mL—millilitres, SD—standard deviation, IQR—interquartile range.

Performance Metric	Surgeon Experience with Fibrin Sealant	Level	Interventional Arm	Control Arm	*p*-Value
Comprehensive Complication Index	Established Users	Mean (SD)	4.5 (10.1)	10.9 (12.6)	0.946
	Learners	Mean (SD)	10.7 (17.7)	8.8 (16.2)	0.740
Total Wound Drainage Volume (mL)	Established Users	Natural Scale Median (IQR)	60 (29, 112)	67 (35, 112)	0.318
	Learners	Natural Scale Median (IQR)	189 (89, 241)	88 (68, 229)	0.760

## Data Availability

Raw data can be requested from the Liverpool Clinical Trials Centre via the following email address: lctc@liverpool.ac.uk.

## Supplementary Materials

The following supporting information can be downloaded at: https://www.mdpi.com/article/10.3390/cancers15205073/s1, Supplementary Data S1. CONSORT Checklist. Supplementary Data S2. Patient Information Sheet. Supplementary Data S3 Missing data and outliers. Supplementary Data S4. Health Economic Analysis.
