# Supplementary material for: Determining the Effectiveness of Fibrin Sealants in Reducing Complications in Patients Undergoing Lateral Neck Dissection (DEFeND): A Randomised External Pilot Trial
Source: Cancers (Basel). 2023 Oct 20;15(20):5073. doi: 10.3390/cancers15205073 (PMC10605578; doi:10.3390/cancers15205073)
Supplement: Supplementary file 1 [file cancers-15-05073-s001.zip › cancers-2575672-supplementary/Supplementary Data S2. Patient Information Sheet.pdf]

PATIENT INFORMATION SHEET

DEFeND

Determining the Effectiveness of Fibrin Sealants in Reducing Complications in Patients Undergoing Lateral Neck Dissection: A randomised external pilot trial

You have been invited to take part in a research study. Before you decide, it is important that you understand why the research is being done and what it will involve. Please take time to read the following information carefully and discuss it with others if you wish.

- **Part One** tells you the purpose of the study and what will happen to you if you take part.
- **Part Two** gives you more detailed information about the conduct of the study.

The clinical team in charge of your care will go through the details with you, but please ask us if there is anything that is not clear or if you would like more information. Take time to decide whether or not you wish to take part.

Thank you for reading this.

Part One

What is the purpose of the study?

A neck dissection is an operation to remove the glands in the neck either because they have cancer in them or they are at risk of cancer spreading to them. Complications after neck dissection can be a significant problem for patients and may affect their quality of life.

Research on understanding the feelings of patients who have had head and neck cancer treatment, has shown that avoiding complications is very important. We have found evidence that by giving patients a substance that copies the blood clotting process called Fibrin Sealant, we may be able to protect them from complications. This is because this substance can seal areas of bleeding and stick the raw surfaces of the wound together, reducing the space for blood to collect in. Fibrin Sealants are natural products derived from the same human blood that is used for blood transfusions and are sprayed directly into the wound. Unfortunately, there is no high quality research that has been able to answer whether Fibrin

Sealants can prevent complications after neck dissection. Therefore we have designed a clinical trial to help us answer this important question. However, before this can be started we need to conduct a miniature version of the trial (pilot study) to make sure it has been designed in the best possible way. Please be aware that this information sheet is for the pilot study and not the full trial. With your help we will be able to improve the design of our future trial to make sure we can find out whether or not Fibrin Sealants can really help avoid complications.

Why have I been chosen?

You were chosen to take part in this study because the team of surgeons and doctors looking after you think that you may require a neck dissection (an operation to remove the glands in your neck). We will be asking all patients who are due to have a neck dissection and meet our criteria for being involved in the study.

Do I have to take part?

No. It is up to you to decide whether or not to take part. If you do, you will be asked to sign a consent form but you are free to withdraw at any time. If you do decide not to take part or withdraw from the study we will be very interested to understand your reasons, however you do not have to disclose this information. **A decision not to take part, or a decision to withdraw at any time will <u>not</u> affect the standard of care you receive**.

What will happen to me if I take part?

If you decide to take part, you will receive all of your treatment as normal. The only differences will be:

1. Before the operation you will be asked to complete a very short questionnaire regarding neck and shoulder function.
2. If you are a woman of childbearing age, you will be offered a pregnancy test. If you are found to be pregnant you will not be allowed to take part in the study.
3. During the operation and in the days following your operation the nurse will lightly wipe your neck and mouth with a cotton buds. This will be used to grow bacteria in a laboratory (University of Liverpool) to increase our understanding of infections after surgery and will not be painful. If you do not wish to have this extra test you can still take part in the rest of the study.
4. At the end of your operation (just before the surgeon starts stitching the wound back together) you will be randomly allocated to either have the Fibrin Sealant sprayed into your wound or not. There is a 50:50 chance you will receive the Fibrin Sealant. Both you and the nurses looking after you will not be told whether you have had the Fibrin Sealant. After the operation you will return to the ward and the doctors and nurses will work hard to get you better as they normally do.
5. Once you have been discharged home, you will need to come back to hospital 1 – 2 weeks later. During this appointment your wounds will be assessed and any stitches/clips removed. The nurse will also take some more cotton buds of your neck wound and mouth.
6. If you need to attend any unscheduled hospital appointments because of a problem/complication, you will need to inform the research team (telephone number on page 5).

7. During an appointment 4 – 6 weeks after the surgery you will re-take the short questionnaire you took before the surgery regarding neck and shoulder function as well as another questionnaire asking you about wound healing and infection. In addition to this you will be asked whether or not you think you received the Fibrin Sealant during your operation and how certain you are of this. This is so we can assess how good we were at keeping it a secret from you. You will also be asked how much benefit the Fibrin Sealant would need to provide before you would consider recommending it to future patients. After this appointment you will exit the study and carry on with the same care that everyone else, who is not in the study, receives.

What do I have to do?

If you take part in this study:

1. You will be asked to take some very short questionnaires before your surgery and at 4 – 6 weeks after your surgery (see points 1 and 7 above).
2. You will need to attend 2 hospital appointments after your surgery. The first 1 – 2 weeks after surgery and the second 4 – 6 weeks after surgery.
3. If you need to attend any unscheduled hospital appointments because of a problem you will need to contact the research team (telephone number on page 5).

Everything else will be done for you and you don't need to take any extra drugs or medicines. There are no restrictions on any further treatment you may need after the surgery. The only extra test will be the cotton bud test to check for bacteria in your mouth and neck but you do not have to agree to this extra test. If you need to attend an extra hospital appointment purely for this research, reasonable travel expenses will be reimbursed.

What is being tested?

Fibrin Sealants are products that are derived from human blood that is used for blood transfusions. They are already being sold on the market and are approved and licenced for use in patients. They work by copying the clotting process and by forming a glue that seals leaking blood vessels and sticks the raw surfaces of the wound together. There is a 50:50 chance that you will have Fibrin Sealant sprayed inside the wound just before the surgeon stitches it back together while you are under anaesthetic. If you do receive the Fibrin Sealant it will only be sprayed once during your surgery.

What are the alternatives for treatment?

The alternatives for treatment are to have your surgery without the Fibrin Sealant.

What are the possible disadvantages and risks of taking part?

Taking part in this study will not have an effect on your cancer treatment. You will not be restricted in taking any drugs you may need or in having any further treatment.

Fibrin Sealants are considered to be very safe products and serious risks are very rare. The manufacturer reports the following potential risks:

1. **Itchiness** of the skin of the neck (between 1 – 10 patients out of a hundred). If you experience itchiness your doctor may treat this with antihistamines (allergy medicines) depending on how problematic this is for you.

2. **Fluid collection** under skin (less than 1 patient out of a hundred). Most fluid collections do not require treatment as your body will eventually absorb the fluid. If the fluid collection is large or problematic, your doctor may drain the fluid. This may be done by either drawing the fluid out using a syringe or formally opening the wound and letting the fluid out.

3. **Severe allergic reaction** (less than 1 patient out of a hundred). If you develop a severe allergic reaction it will normally develop during or immediately after the surgery. If signs and symptoms of allergy are noted you will receive treatment as a matter of urgency.

4. If the surgeon holds the spray too close to the blood vessels in your neck, air may enter the blood vessel causing a serious complication known as an **"air embolism"**. Patients who get "air embolisms" are at increased risk of heart attacks, strokes and breathing problems. Fortunately this is very rare as there have only ever been **6 reported cases** of life threatening "air embolism" out of many thousands of patients who have already been given Fibrin Sealants over the years. There have been no reported cases of "air embolism" from the type of Fibrin Sealant we intend to use (ARTISS, Baxter Healthcare LTD). **Every surgeon who uses the fibrin sealant in this study will be trained on how to avoid this complication**.

5. Because the Fibrin Sealant is taken from human blood that is used in blood transfusions, there is a **theoretical risk** of catching a **blood borne virus** (e.g. Hepatitis or HIV). People who donate their blood are always carefully selected to minimise the risk of transmitting viruses. Also the Fibrin Sealant has been carefully checked and treated to prevent contamination with viruses. Despite these efforts, we cannot guarantee that the Fibrin Sealant is free of viruses. There have been **no reported cases** of patients catching viruses from Fibrin Sealants in the scientific literature. In the very unlikely event that you do catch a virus, you will be referred to a specialist for treatment.

What are the possible benefits of taking part?

There are no specific benefits to taking part other than giving you the opportunity to take part in surgical research. The study has been designed to minimise extra tests and hospital visits so that your participation is as easy for you as possible.

Surgeons up and down the country are already using Fibrin Sealants however this is not based on high quality evidence. By participating in this pilot study, you will be helping us to design a 'full' trial that is as effective and efficient as possible. We hope that the 'full' trial will enable us to answer whether or not Fibrin Sealants are beneficial to patients undergoing neck dissection surgery.

Will my taking part in this study be kept confidential?

Yes. All the confidential information about your participation in this study will be treated as so. The detailed information on this is given in Part 2.

What if I wish to make a complaint?

Any complaint about the way you have been dealt with during the study or any possible harm you might suffer will be addressed. If you wish to raise some informal concerns or complaints please contact the Research Team (contact details below). If you wish to make a formal complaint about any aspect of this study please do this through the University of Liverpool Research Integrity and Governance Manager (contact details below). Further details on the complaints procedure can be found in Part Two under *"what if there is a problem?"*

For further information please contact the Research Team:

Appropriate job title:

[Trust to insert contact details]

Contact Number is:

[Trust to insert contact details]

For any complaints please contact the Research Integrity and Governance Manager:

Research Integrity and Governance Manager
Research Support Office
University of Liverpool / Liverpool Joint Research Office
2nd Floor Block D Waterhouse Building
3 Brownlow Street
Liverpool L69 3GL

Tel: 0151 794 8373
Email: sponsor@liverpool.ac.uk

This completes Part One of the Information Sheet. If the information in Part One has interested you and you are considering participation, please continue to read the additional information in Part Two before making any decision.

Part Two

What if new information becomes available?

Sometimes during the course of a research project, new information becomes available about the treatment that is being studied. If this happens, your doctor will tell you about it and discuss with you whether you want to or should continue in the study. If you decide to withdraw your doctor will make arrangements for your care to continue. If you decide to continue in the study you will be asked to sign an updated consent form.

On receiving new information your doctor might consider it to be in your best interests to withdraw you from the study. He/she will explain the reasons and arrange for your care to continue.

If the study is stopped for any other reason you will be told why and your continuing care will be arranged.

What will happen if I don't want to carry on with the study?

If you no longer wish to take part in the study you can withdraw your consent at any time. We would ask that you contact us using the contact details written at the end of section one. We would be very interested to understand why you decided to withdraw from the study but you do not have to tell us. Any feedback you provide will be confidential and only used to try to improve the way the study is run.

Once you have left the study you will simply carry on with the treatment and hospital appointments that patients who are not involved in the study will receive. Regarding the information and samples we collected for the study, it is your choice if we keep them or discard them securely. We will clarify this with you at the time. Any safety information collected (complications that occur after surgery) however cannot be discarded and will be used.

What if there is a problem?

If you have a concern about any aspect of this study, you may speak with the Research Team at your hospital (contact details at end of Part One) who will do their best to answer your questions. If they are unable to answer your question, they may wish to escalate the query to the Chief Investigator or North West Surgical Trials Centre (University of Liverpool) who are responsible for overseeing the study. If you remain unhappy and wish to complain formally, you can do this through the University of Liverpool's Research Integrity and Governance Manager (contact details at end of Part One)

If you are harmed by taking part in this research project, there are no special compensation arrangements. If you are harmed and this is due to someone's negligence, then you may have grounds for a legal action for compensation against the NHS Trust where you are being treated, but you may have to pay for your legal costs. The normal National Health Service complaints mechanisms should be available to you (if appropriate).

In the event of defective product then you may have grounds for a legal action for compensation against the manufacturer, but you may have to pay for your legal costs.

Will my taking part in this study be kept confidential?

If you join the study, some relevant parts of your medical records and the data collected for the study will be looked at by authorised persons from the North West Surgical Trials Centre (University of Liverpool) or their collaborators who are also involved in organising this research project. Data may also be looked at by representatives of regulatory authorities and by authorised people from the Trust or other NHS bodies to check that the study is being carried out correctly. **All will have a duty of confidentiality to you as a research participant**.

A scanned copy of your completed consent form will be securely uploaded to the North West Surgical Trials Centre (University of Liverpool) portal. Only delegated members of the research team will have access to this portal. The uploaded consent form will be checked by the Trial Manager based at the University of Liverpool to ensure it has been completed correctly. Once this check has been performed the electronic copy of your consent form will be permanently deleted. This means that the hospital that is treating you will hold your original consent form and no other copies will be stored elsewhere.

Hard copies of your consent form and any other hard copies of data collected for this study will be stored in your medical records. No hard copies of your data will be stored at the University of Liverpool. The electronic research data gathered will contain your initials, date of birth and NHS number. This research data will be stored for 15 years within a secure file held within the University of Liverpool.

Involvement of the General Practitioner/Family Doctor (GP)

With your consent, your GP will be informed of your involvement in the study. Any other medical practitioners who treat you, should you be admitted to hospital for any reason, will also be informed.

What will happen to any samples I give?

With your permission, we would like to transfer cotton bud swabs of your mouth and wound to the University of Liverpool for storage. These samples will be used only for investigating surgical infections and will not be used for any commercial purposes.

The samples will be kept in a secure place until we need them; nobody outside of the study will have access to **any** confidential information that you give to us. Confidential details (such as your name, address and GP details) will be kept locally and not made available to collaborators.

Your sample will be coded and the researchers carrying out tests on the samples will not be given information they do not need to carry out the tests and analyse the results. Coded is not the same as anonymous. It will be possible to use the codes to identify that a result is from your sample. However, we do not plan to do this unless there is a good reason to do so. We will maintain this information so that we can properly manage the samples donated. For instance, sometimes we may need to update our record of your clinical details to help us interpret the results of tests.

All of your samples will be destroyed at the end of the study

Will any genetic tests be done?

Only on the bacteria that we grow from your wound swab. We will not carry out any genetic tests on human tissue.

What will happen to the results of the research study?

It is intended that once the study is complete a report will be written and the results will be published to make them available to the public. You will not be named or identified in any publication.

What rights do I have to the results of the research?

Any information derived directly or indirectly from this research, as well as any patents, diagnostic tests, drugs, or biological products developed directly or indirectly as a result of this research may be used for commercial purposes. You have no right to this property or to any share of the profits that may be earned directly or indirectly as a result of this research. However, in signing this form you do not give up any rights that you would otherwise have as a participant in research.

Who is organising and funding the research?

The research is being organised by the North West Surgical Trials Centre which is part of the University of Liverpool in collaboration with Aintree University Hospital in Liverpool. The research is being funded by the National Institute of Health Research which is part of the Department of Health (UK). The data collected in this study will contribute towards a PhD for a student based in the University of Liverpool.

Your doctor will not receive any payment for including you in this study.

Who has reviewed the study?

The study has been reviewed for scientific content by members of the **North West – Greater Manchester East Research Ethics Committee**.

Thank you for taking the time to read and consider this information sheet. Should you decide to take part in the study, you will be given a copy of the information sheet and a signed consent form to keep.
