# Supplementary material for: Determining the Effectiveness of Fibrin Sealants in Reducing Complications in Patients Undergoing Lateral Neck Dissection (DEFeND): A Randomised External Pilot Trial
Source: Cancers (Basel). 2023 Oct 20;15(20):5073. doi: 10.3390/cancers15205073 (PMC10605578; doi:10.3390/cancers15205073)
Supplement: Supplementary file 1 [file cancers-15-05073-s001.zip › cancers-2575672-supplementary/Supplementary Data S3 Missing data and outliers.pdf]

SUPPLEMENTARY DATA S3. MISSING DATA AND POSSIBLE OUTLIERS

Summary of missing data by treatment arm

	Variable	Interventional arm	Control arm	Unassigned	Total
Baseline Demographics	**Total**	**26**	**25**	**2**	**53**
	Hb	3 (12%)	2 (8%)	0 (0%)	5 (10%)
	Platelets	3 (12%)	2 (8%)	0 (0%)	5 (10%)
	White cells	3 (12%)	3 (12%)	0 (0%)	6 (12%)
	PT	9 (35%)	8 (32%)	1 (50%)	17 (33%)
	aPTT	10 (38%)	8 (32%)	1 (50%)	18 (35%)
	**Total**	**25**	**23**		**48**
Drain	Drain volume	0 (0%)	1 (4%)	-	1 (2%)
NDII	Follow-up 2	1 (4%)	3 (13%)	-	4 (8%)
Surgery	Cutting Instrument	0 (0%)	1 (4%)	-	1 (2%)
	Blood Loss	0 (0%)	1 (4%)	-	1 (2%)
	Length of Surgery	2 (8%)	3 (13%)	-	5 (10%)
	Time to Reveal	0 (0%)	2 (9%)	-	2 (4%)
	Time to Recovery Room	2 (8%)	2 (9%)	-	4 (8%)

Summary of missing data by site

	Variable	Queen Victoria Hospital	Aintree University Hospital	Total
Baseline Demographics	**Total**	**7**	**46**	**53**
	Hb	2 (29%)	3 (7%)	5 (10%)
	Platelets	2 (29%)	3 (7%)	5 (10%)
	White cells	3 (43%)	3 (7%)	6 (12%)
	PT	7 (100%)	11 (24%)	18 (33%)
	aPTT	7 (100%)	12 (26%)	19 (35%)
	**Total**	**6**	**42**	**48**
Drain	Drain volume	0 (0%)	1 (2%)	1 (2%)
NDII	Follow-up 2	1 (17%)	3 (7%)	4 (8%)
Surgery	Cutting Instrument	0 (0%)	1 (2%)	1 (2%)
	Blood Loss	0 (0%)	1 (2%)	1 (2%)
	Length of Surgery	1 (17%)	4 (10%)	5 (10%)
	Time to Reveal	0 (0%)	2 (5%)	2 (4%)

Summary of possible outliers by treatment arm

	Covariate	Severity	Interventional arm	Control arm	Total
	Total		25	23	48
Drain	Volume	Mild	0 (0%)	1 (4%)	1 (2%)
Surgical Outcomes	Blood Loss	Mild	2 (8%)	4 (17%)	6 (13%)
		Severe	2 (8%)	0 (0%)	2 (4%)
	Length of Surgery	Mild	0 (0%)	1 (4%)	1 (2%)
		Severe	1 (4%)	0 (0%)	1 (2%)
	Time to Reveal	Mild	0 (0%)	1 (4%)	1 (2%)
		Severe	0 (0%)	1 (4%)	1 (2%)
