# Supplementary material for: Determining the Effectiveness of Fibrin Sealants in Reducing Complications in Patients Undergoing Lateral Neck Dissection (DEFeND): A Randomised External Pilot Trial
Source: Cancers (Basel). 2023 Oct 20;15(20):5073. doi: 10.3390/cancers15205073 (PMC10605578; doi:10.3390/cancers15205073)
Supplement: Supplementary file 1 [file cancers-15-05073-s001.zip › cancers-2575672-supplementary/Supplementary Data S4. Health Economic Analysis.pdf]

SUPPLEMENTARY DATA S4. Health economic analysis

Relevant resource events for each patient were extracted from the CRF into a Microsoft Excel workbook. The extracted information included appointments with healthcare professionals, procedures performed, inpatient hospitalisation, and management of adverse events. Data on community healthcare resource use (e.g. district nurses, GP appointments) and patient absenteeism from work were not collected.

Resource Use

1. Consultant clinic appointment as screening for trial
2. Day of surgery
3. Excess bed-days
4. Returns to theatre
5. Post-operative outpatient attendance, including unscheduled visits

Time in theatre

As National Health Service (NHS) Reference Costs include the costs of theatre time in their estimates, adding additional costs based on time in surgery would double-count this element of resource use. Therefore, no costs have been added to the ‘time in surgery’ field.

The analysis was based on 2019/20 NHS tariffs.

Table S1: Unit cost of resource use items

Resource	Description	Unit cost	Source
**Outpatient**			
Maxillo-Facial Surgery first visit	Maxillo-Facial Surgery attendance – single professional; HRG code: WF01B	£147	NHS tariff
Maxillo-Facial Surgery follow-up visit	Maxillo-Facial Surgery attendance – single professional; HRG code: WF01A	£66	NHS tariff
ENT first visit	ENT attendance – single professional; HRG code: WF01B	£121	NHS tariff[ref]
ENT follow-up visit	ENT attendance – single professional; HRG code: WF01A	£52	NHS tariff[ref]
**Surgery**			
T851 Block dissection of cervical lymph nodes	Major Neck Procedures with CC Score 0-1. Combined day and elective spell; HRG code: CA03B. Ordinary elective long stay trim point = 5 days	£3,157	NHS tariff[ref]
F382 Excision of lesion of mouth NEC; T851 Block dissection of cervical lymph nodes	Major Neck Procedures with CC Score 0-1. Combined day and elective spell; HRG code: CA03B. Ordinary elective long stay trim point = 5 days	£3,157	NHS tariff[ref]
E231 Open excision of lesion of pharynx; T851 Block dissection of cervical lymph nodes	Very Complex, Mouth or Throat Procedures, with CC Score 0-1. Combined day and elective spell; HRG code: CA80C. Ordinary elective long stay trim point = 26 days	£7,504	NHS tariff
T851 Block dissection of cervical lymph nodes; T81.0 Haemorrhage and haematoma complicating a procedure, not elsewhere classified	Major Neck Procedures with CC Score 2+. Combined day and elective spell; HRG code: CA03A. Ordinary elective long stay trim point = 6 days	£3,815	NHS tariff
F382 Excision of lesion of mouth NEC; T851 Block dissection of cervical lymph nodes; T81.0 Haemorrhage and haematoma complicating a procedure, not elsewhere classified	Major Neck Procedures with CC Score 2+. Combined day and elective spell; HRG code: CA03A. Ordinary elective long stay trim point = 6 days	£3,815	NHS tariff
E231 Open excision of lesion of pharynx; T851 Block dissection of cervical lymph nodes; T81.0 Haemorrhage and haematoma complicating a procedure, not elsewhere classified	Very Complex, Mouth or Throat Procedures, with CC Score 2-4. Combined day and elective spell; HRG code: CA80B. Ordinary elective long stay trim point = 32 days	£11,315	NHS tariff
Excess bed-day	Additional bed-day beyond ordinary elective long stay trim point	£265	NHS tariff
Artiss fibrin sealant	Combined cost of product and disposables	£98.23	Baxter Healthcare

ENT= Ear, Nose & Throat; HRG=health resource group

Table S2: Number of resource use episodes and mean resource use per patient

Resource	Interventional Arm (n=25)			Control Arm (n=23)		
	No. patients	No. episodes	Episode per patient*	No. patients	No. episodes	Episode per patient*
**Outpatient**						
Maxillo-Facial Surgery first visit	25	6	0.240	23	9	0.391
Maxillo-Facial Surgery follow-up visit	25	18	0.720	23	30	1.304
- *Follow-up 1*	*25*	*6*	*0.240*	*23*	*9*	*0.391*
- *Follow-up 2*	*25*	*6*	*0.240*	*23*	*9*	*0.391*
- *Unscheduled visit*	*25*	*6*	*0.240*	*23*	*12*	*0.521*
ENT first visit	25	19	0.760	23	14	0.609
ENT follow-up visit	25	46	1.840	23	42	1.826
- *Follow-up 1*	*25*	*19*	*0.760*	*23*	*14*	*0.609*
- *Follow-up 2*	*25*	*18*	*0.720*	*23*	*12*	*0.521*
- *Unscheduled visit*	*25*	*9*	*0.360*	*23*	*16*	*0.696*
**Surgery**						
T851 Block dissection of cervical lymph nodes	25	10	0.400	23	9	0.391
F382 Excision of lesion of mouth NEC; T851 Block dissection of cervical lymph nodes	25	1	0.040	23	2	0.087
E231 Open excision of lesion of pharynx; T851 Block dissection of cervical lymph nodes	25	12	0.480	23	10	0.435
T851 Block dissection of cervical lymph nodes; T81.0 Haemorrhage and haematoma complicating a procedure, not elsewhere classified	25	0	0.000	23	1	0.043
F382 Excision of lesion of mouth NEC; T851 Block dissection of cervical lymph nodes; T81.0 Haemorrhage and haematoma complicating a procedure, not elsewhere classified	25	1	0.040	23	0	0.000
E231 Open excision of lesion of pharynx; T851 Block dissection of cervical lymph nodes; T81.0 Haemorrhage and haematoma complicating a procedure, not elsewhere classified	25	1	0.040	23	1	0.043
Additional bed days	25	1	0.040	23	1	0.043
Artiss	25	25	1.000	23	0	0.000
Number of complications	25	10	0.400	23	14	0.609

*=values have been reweighted to include all patients in the treatment arm.

Table S3: Mean cost per patient per treatment arm

Resource	Interventional Arm (n=25)	Control Arm (n=23)
**Outpatient**		
Maxillo-Facial Surgery first visit	£35.52	£57.48
Maxillo-Facial Surgery follow-up visit	£47.52	£86.06
ENT first visit	£91.96	£73.69
ENT follow-up visit	£95.68	£94.95
Total outpatient cost per patient	***£270.68***	***£312.18***
**Surgery**		
T851 Block dissection of cervical lymph nodes	£1,282.80	£1,234.39
F382 Excision of lesion of mouth NEC; T851 Block dissection of cervical lymph nodes	£126.28	£274.66
E231 Open excision of lesion of pharynx; T851 Block dissection of cervical lymph nodes	£3,601.92	£3,264.24
T851 Block dissection of cervical lymph nodes; T81.0 Haemorrhage and haematoma complicating a procedure, not elsewhere classified	£0	£164.05
F382 Excision of lesion of mouth NEC; T851 Block dissection of cervical lymph nodes; T81.0 Haemorrhage and haematoma complicating a procedure, not elsewhere classified	£152.60	£0
E231 Open excision of lesion of pharynx; T851 Block dissection of cervical lymph nodes; T81.0 Haemorrhage and haematoma complicating a procedure, not elsewhere classified	£452.60	£486.55
Total surgery cost per patient	***£5616.20***	***£5423.89***
Additional bed days	£10.60	£11.52
Artiss	98.23	0
Total cost per patient	***5,995.71***	***£5,747.59***

Incremental Cost Effectiveness Ratio (ICER) Calculations

Resource	Interventional Arm (n=25)			Control Arm (n=23)		
	No. patients	No. episodes	Episode per patient*	No. patients	No. episodes	Episode per patient*
Complication	25	10	0.400	23	14	0.609
Bed days	25	73	2.920	23	78	3.391
Length of surgery (median)	25		2.467	23		2.050

Difference in cost per patient

The Interventional arm costs £248.12 more per patient than the control arm.

Difference in complications

The control arm had 0.209 more complications per patient than the interventional arm.

Difference in bed days

Patients in the control arm stayed 0.471 days per patient longer than the interventional arm.

Difference in length of surgery

Patients in the interventional arm took a median of 0.417 hours (approximately 25 minutes) longer in surgery than in the control arm.

Complications ICER = £248.12 / 0.209

= £1,187.17 per complication prevented

Bed days ICER = £248.12 / 0.471

= £526.79 per bed day saved

FS costs more, does more and lies in the upper right quadrant of the ‘cost-effectiveness plane’. The origin of the ‘cost-effectiveness plane’ is the control arm. Based on an excess bed day valued at £265, a cost of £526.79 per bed day saved does not seem cost-effective. It is also noted that patients in interventional spent a longer time in surgery.

The excess costs due to complications in this study manifested as unscheduled outpatient visits, higher NHS tariffs because of returns to theatre and excess bed days. On an individual patient level, some minor complications did not incur any financial cost, whereas the most severe complication incurred an excess cost of £3,915. Whether the complication ICER of £1,187.17 per complication prevented represents, value for money is difficult to answer. This is because complications are associated with physical, psychological and social costs to the patient that has not been quantified in this analysis. One must accept that this calculation is fundamentally flawed as it is based on NHS tariffs and does not consider how quickly patients returned to work or their usage of community healthcare. On this basis, no firm conclusions can be made regarding the cost-effectiveness of FS. A definitive RCT should include patient diaries to record absenteeism from work and community healthcare usage. Patients should also be followed up for longer so that they may complete the EQ-5D questionnaire to enable the calculation of ICER based on quality-adjusted life years (QALYs). If patients are followed up for longer, adjuvant treatment (RT/CRT) costs would also need to be included in the analysis. This would undoubtedly “muddy the water” but is justified within a randomised pragmatic trial design.
